# Efficacy of insecticide-infused hydrogel baits in managing European earwig (Dermaptera: Forficulidae) in citrus

**DOI:** 10.1093/jee/toag108

**Published:** 2026-04-28

**Authors:** Sanjeev K Dhungana, Sandipa G Gautam

**Keywords:** citrus, European earwig, spinosad, thiamethoxam

## Abstract

European earwig (*Forficula auricularia* L.) is an omnivorous insect commonly found in California citrus orchards. Often considered a beneficial predator, earwigs can damage young fruit, causing rind scarring and reducing the market value. Moreover, earwig feeding can cause defoliation of young citrus trees, thereby creating the need to manage the population. We conducted laboratory and field trials in 2024 and 2025 to evaluate the efficacy of insecticide-infused hydrogel baits for earwig management. Polyacrylamide hydrogel beads were soaked in thiamethoxam or spinosad solutions at 0.1×, 0.5×, and 1× of label rate (*x*). In the first laboratory trial, ∼3 cm^3^ of treated hydrogel prepared with 25% (w/v) added sucrose was placed in plastic containers with field-collected earwigs, with or without lemon leaves (choice and no-choice tests). The results of choice and no-choice tests showed that earwig mortality increased over time (12 to 144 h) across all concentrations and insecticides in both tests compared to the untreated check. For the field trial, ∼20 cm^3^ of hydrogel with added sucrose was applied per tree into the trunk wrap. The number of earwigs per tree was significantly reduced in all insecticide treatments compared to the untreated check at 6- and 14-d post-treatment. In the second laboratory trial, no significant difference in mortality was observed in bait with or without added sucrose, with mortality ranging 83.1% to 100% at 144 h. The results show that insecticide-infused hydrogel baits, regardless of added sucrose, can be a promising tool for managing earwigs in citrus orchards.

## Introduction

California is a major region for fresh citrus production, accounting for 79% of the total US citrus production (USDA 2023). Any kind of rind damage significantly decreases market value as California citrus targets fresh markets. Extensive feeding by any pest on young fruit can cause scars when the fruit matures (Cass et al. 2019, Grafton-Cardwell et al. 2020). European earwigs (*Forficula auricularia* L.) are omnivores in crop systems. Increased earwig infestations in citrus orchards may be related to reduced use of broad-spectrum insecticides (Logan et al. 2011, Romeu-Dalmau et al. 2012a). Earwigs can be pests as they defoliate young trees and cause damage to fruit (Kahl et al. 2022). However, they are also beneficial, as they prey on several pest species like California red scale (Romeu-Dalmau et al. 2012a) and aphids (Piñol et al. 2009, Romeu-Dalmau et al. 2012b).

Research on earwigs in citrus has mainly focused on their beneficial role, while very little has reported on their damaging role (Romeu-Dalmau et al. 2012a; Orpet et al. 2019). One study revealed that earwigs restricted to citrus branch terminals caused significant harm to young navel oranges (Kahl et al. 2022). Fruits that experienced substantial earwig damage either fell off prematurely or developed noticeable scars as they grew (Kahl et al. 2021), likely resulting in downgrading at the packinghouse and reduced grower profits. California citrus researchers and farm advisors have reported that earwigs can chew holes and create scars in young citrus fruits (Kahl et al. 2023).

Earwigs are reported to cause fruit damage in citrus by feeding on small, developing fruits in one to four weeks immediately following petal fall (Grafton-Cardwell et al. 2020). Additionally, earwigs can defoliate young trees with trunk wraps or cardboard guards as they hide in the wraps, climb the trees, and feed on the new leaves (Grafton-Cardwell et al. 2020).

The extent of earwig damage to citrus was first reported in detail by University of California researchers. Earwigs can cause damage to multiple citrus varieties, including navel oranges, clementines, and true mandarins, and the varieties vary in their susceptibility (Kahl et al. 2022, 2022, 2023). Other studies have focused on monitoring and managing earwigs using cardboard traps (Helsen et al. 1998, Orpet et al. 2019, DuPont et al. 2023, Hanel et al. 2023) and poisoned baits (Fulton 1923). Chemical management, soil or foliar application, can be challenging as the nocturnal nature of this pest makes it difficult to monitor populations for timely application. Other chemical options available are spinosad baits. The use of insecticide-infused hydrogel beads to control ants in agricultural cropping systems in California has been reported by several researchers (Rust et al. 2015, McCalla et al. 2020, Le et al. 2024, Milosavljević et al. 2024). In this study, we evaluated the efficacy of insecticide-infused hydrogel baits under choice and no-choice laboratory assays against European earwigs. The second objective was to assess bait efficacy with and without the addition of sugar. In addition, a field trial was conducted to validate laboratory results under commercial orchard conditions.

## Materials and Methods

Laboratory and/or field experiments were conducted in 2024 and 2025 at the Lindcove Research and Extension Center in Exeter, California.

### Laboratory Bioassay Using Hydrogel Bait with Added Sucrose in 2024

The first laboratory trial was conducted to test the efficacy of three rates of Actara 25WG (a.i. thiamethoxam, manufacturer: Syngenta) and Entrust 2SC (a.i. spinosad, manufacturer: Corteva Agriscience) on the mortality of European earwigs using hydrogel as a delivery medium to dispense the insecticide. Three concentrations, 0.1× (low), 0.5× (medium), and 1× (high) of the label rate (*x*) were tested. Insecticide-infused hydrogel beads for each treatment were prepared by first making 1 gallon (3.785 liter) insecticide solution in 25% (w/v) sucrose (Table sugar, Great Value). Then, 49.5 g dry polyacrylamide hydrogel beads (Soil Moist, JRM Chemical, Cleveland, Ohio) were added to each insecticide solution and allowed to soak for 16 h. Efficacy was evaluated in the presence of a food source (choice test) and without a food source (no-choice test), using field-collected immature earwigs. Foam wraps wrapping the trunk of 1-yr-old citrus trees were gently lifted and tapped to dislodge earwigs onto a piece of cardboard (∼25 cm × 25 cm). The earwigs were quickly transferred to plastic containers with modified lids fitted with a fabric mesh allowing air exchange and transported to the laboratory. This newly planted field, where earwigs were collected, was not exposed to insecticides. Moreover, we did not find any sign of contamination with entomopathogens in the field-collected earwigs during the experimental period. Earwigs used in the experiment uniformly comprised second and third instars. European earwigs overwinter as adults, immatures hatch and develop in spring, and populations are synchronized, consisting of immatures in early spring. For each treatment, ∼10 earwigs were released into plastic containers (11 cm in diameter and 4 cm deep) with modified lids fitted with fabric mesh for air circulation. The choice test included 2 to 3 tender lemon leaves and ∼3 cm^3^ of hydrogel, lodged to the sides of a container, leaving 2 to 3 cm space in between; the leaves were replaced every 24 h. The no-choice test only contained ∼3 cm^3^ of hydrogel. The experimental design was a completely randomized design (CRD), with 10 containers as replicates. Environmental conditions in the laboratory were maintained at a temperature of 22 ± 2 °C, relative humidity of 60 ± 10%, and a cycle of 14 h of light and 10 h of dark. Post-treatment assessments were made by counting the number of live and dead earwigs at 2, 4, 8, 12, 24, 48, 72, and 144 h, and percent mortality was calculated.

### Laboratory Bioassay Using Hydrogel Bait With and Without Added Sucrose in 2025

A second laboratory trial was conducted in 2025 to evaluate whether adding sucrose was necessary as an attractant for the efficacy of Actara and Entrust on European earwig mortality. The bioassay was done using hydrogel bait prepared with and without 25% (w/v) sucrose, following a similar protocol as described above. As in 2024, the field and trees, where earwigs were collected, were not exposed to insecticides, and we did not find any sign of contamination with entomopathogens in the field-collected earwigs during the experiment period. The number of live and dead earwigs was recorded at 2, 4, 8, 12, 24, 48, 72, and 144 h after exposure to the hydrogel baits, and the percent mortality was calculated.

### Field Experiment Using Hydrogel Bait With Added Sucrose in 2024

A field trial was carried out in a 1-yr-old “Tango” mandarin orchard to evaluate the effectiveness of low, medium, and high concentrations, ie 0.1×, 0.5×, and 1× of the label rate, respectively, of Actara and Entrust applied using insecticide-infused hydrogel. The experiment followed a CRD, with 10 individual trees as replicates. A pretreatment evaluation was conducted on 6 March by gently lifting and tapping the trunk wrap of each tree and recording the number of earwigs present. The field had a somewhat uniform earwig presence; therefore, nearly all trees were infested. To minimize variability due to earwig population among treatments, we discarded trees with no earwigs or with too high counts during the pretreatment count. Insecticide-infused hydrogel beads were prepared with sucrose using the method described above in the first trial. On 19 March, the hydrogel bait was applied to the trees by pouring ∼20 cm^3^ (one heaping tablespoon) of hydrated gel beads inside each tree’s trunk wrap. The untreated check received hydrogel soaked in the sucrose solution only. Post-treatment assessments were conducted using the same method as the pretreatment evaluation at 2, 6, and 14 days after treatment (DAT).

### Data Analysis

Mortality counts were taken over time from the same experimental units, and cumulative mortality for each assessment interval (eg 2, 4, 8, 12, 24, 48, 72, and 144 h) was analyzed separately. The experimental unit for the field trial was a single tree with its trunk wrap. The number of earwigs per tree was counted 12 d before treatment (pre-treatment count) and at 2, 6, and 14 d after treatment (post-treatment counts). Data for each count were analyzed separately. Proportion mortality data from the laboratory trial were subjected to arcsine-square root transformation, and count data from the field trial were transformed using log_10_(*x* + 1) before analysis. Data for all experiments were subjected to analysis of variance (ANOVA) using the aov() function in R version 4.4.2 (R Core Team 2024) via RStudio version 2024.12.0 (RStudio Team 2024). For all analyses, treatment means were separated using Fisher’s protected least significant difference (LSD) test at *P *< 0.05.

## Results

### Lab Experiment Using Hydrogel Bait With Added Sucrose

We did not observe significant differences in earwig mortality between choice and no-choice tests across the three concentrations tested, except for treatments with Entrust 1*x* and Actara 0.5*x* (Table 1). From 2 to 24 h after treatment (HAT), mortality was significantly higher in the no-choice test for Entrust 1*x* compared to the choice test at the same concentration. Similarly, for Actara 0.5*x*, mortality was significantly higher in the no-choice test than in the choice test at 8 HAT (Table 1). For all concentrations, mortality increased over time in both cases, regardless of fresh leaves provided as a food source, compared to the untreated check at 12 HAT and beyond. All treatments except Entrust 0.1× resulted in 98% to 100% mortality at 144 HAT. In the untreated check group, no earwig mortality was recorded until 24 HAT in the no-choice test and 144 HAT in the choice test (Table 1), suggesting that hydrogel alone do not harm earwigs.

**Table 1.** Percent mortality of European earwigs at different concentrations and time under choice and no-choice tests in the laboratory (Dhungana and Gautam 2025b)

Treatment/formulation	Concn of active ingredients (%)	Test^a^	Mean % mortality of earwigs at hours after treatment (HAT)							
			2	4	8	12	24	48	72	144
**Check**	-	Choice	0.0 ± 0.0c	0.0 ± 0.0d	0.0 ± 0.0c	0.0 ± 0.0 g	0.0 ± 0.0 g	0.0 ± 0.0f	0.0 ± 0.0e	7.3 ± 3.0c
		No-choice	0.0 ± 0.0c	0.0 ± 0.0d	0.0 ± 0.0c	0.0 ± 0.0 g	1.7 ± 1.7 g	3.7 ± 2.5f	7.3 ± 3.0d	9.0 ± 3.0c
**Entrust 2SC 0.1**×	0.019531	Choice	3.1 ± 2.1bc	7.4 ± 4.4abcd	7.4 ± 4.4bc	29.7 ± 7.2ef	36.0 ± 5.2f	46.4 ± 5.0e	72.8 ± 6.0c	84.8 ± 7.2 b
		No-choice	0.0 ± 0.0c	4.2 ± 2.2bcd	5.9 ± 2.4bc	31.1 ± 5.2ef	40.1 ± 6.0f	49.8 ± 5.9e	71.3 ± 5.4c	83.1 ± 4.4 b
**Entrust 2SC 0.5**×	0.097656	Choice	6.0 ± 3.1 b	11.1 ± 4.3ab	11.1 ± 4.3 b	54.7 ± 6.8 cd	65.5 ± 5.9de	79.6 ± 6.2 cd	94.9 ± 2.7ab	98.0 ± 2.0a
		No-choice	4.8 ± 2.4bc	7.5 ± 3.6abc	9.9 ± 3.7 b	44.2 ± 9.2de	52.7 ± 9.2ef	73.1 ± 5.6d	91.4 ± 4.9 b	100.0 ± 0.0a
**Entrust 2SC 1**×	0.195312	Choice	1.7 ± 1.7bc	1.7 ± 1.7 cd	4.8 ± 2.4bc	73.6 ± 5.3 b	75.6 ± 4.1bcd	89.3 ± 3.8bc	98.3 ± 1.7ab	100.0 ± 0.0a
		No-choice	17.6 ± 5.7a	17.6 ± 5.1a	22.6 ± 6.4a	89.0 ± 3.2a	92.4 ± 3.3a	95.2 ± 2.4ab	98.3 ± 1.7ab	100.0 ± 0.0a
**Actara 25WG 0.1**×	0.011442	Choice	1.7 ± 1.7bc	1.7 ± 1.7 cd	8.3 ± 2.8 b	40.4 ± 4.9de	45.3 ± 4.2f	92.0 ± 4.6a	95.2 ± 3.5ab	100.0 ± 0.0a
		No-choice	2.0 ± 2.0bc	2.0 ± 2.0 cd	5.4 ± 4.1bc	25.0 ± 6.4f	49.1 ± 2.6ef	97.1 ± 2.9a	100.0 ± 0.0a	100.0 ± 0.0a
**Actara 25WG 0.5**×	0.057209	Choice	0.0 ± 0.0c	2.8 ± 1.9bcd	9.4 ± 3.6 b	57.0 ± 8.0bcd	73.3 ± 5.4bcd	100.0 ± 0.0a	100.0 ± 0.0a	100.0 ± 0.0a
		No-choice	0.0 ± 0.0c	2.7 ± 1.8bcd	21.2 ± 3.0a	54.3 ± 4.6 cd	72.1 ± 3.4 cd	98.0 ± 2.0a	100.0 ± 0.0a	100.0 ± 0.0a
**Actara 25WG 1**×	0.114418	Choice	1.7 ± 1.7bc	9.5 ± 3.3ab	32.2 ± 4.3a	72.7 ± 6.8ab	81.3 ± 4.3bc	98.3 ± 1.7a	100.0 ± 0.0a	100.0 ± 0.0a
		No-choice	3.1 ± 2.1bc	7.4 ± 2.5abc	28.8 ± 3.5a	69.0 ± 7.5bc	85.4 ± 3.9ab	100.0 ± 0.0a	100.0 ± 0.0a	100.0 ± 0.0a
***P-*value**			<0.001	0.003	<0.001	<0.001	<0.001	<0.001	<0.001	<0.001
***F-*statistic (df = 13,126)**			3.6	2.6	8.6	22.5	38.7	74.5	95.9	104.0

Means ± SE within each column followed by different letters are significantly different (*P *< 0.05). Fisher’s protected least significant difference (LSD) test was used in mean separation. The percentage mortality of earwigs was arcsine (square root (*x*)) transformed before analysis. Untransformed means are reported.

0.1×: 10% of label rate; 0.5×: 50% of label rate; and 1×: label rate.

a No-choice test consisted of hydrogel baits only, and the choice test consisted of hydrogel baits and lemon leaves.

### Lab Experiment Using Hydrogel Bait With and Without Added Sucrose

There were no significant differences in earwig mortality between treatments with and without sugar after 12 HAT across the 3 concentrations tested, except for Actara 0.1× from 24 to 72 HAT (Table 2). We observed higher mortality in all treatments with sugar from 2 to 12 HAT; however, these differences did not continue with the exposure period. For all concentrations, mortality increased over time in both with and without sugar tests, surpassing the untreated check at 4 HAT and beyond. In the untreated check group, no earwig mortality was recorded until 72 HAT in the treatment with sugar, and until 144 HAT in the treatment without sugar test (Table 2).

**Table 2.** Percent mortality of European earwigs at different concentrations and time with and without sugar in hydrogel bait in the laboratory

Treatment/formulation	Concn of active ingredients (%)	Test1	Mean % mortality of earwigs at hours after treatment (HAT)							
			2	4	8	12	24	48	72	144
**Check**	-	With sugar	0.0 ± 0.0f	0.0 ± 0.0f	0.0 ± 0.0f	0.0 ± 0.0e	0.0 ± 0.0d	0.0 ± 0.0c	1.3 ± 0.0c	1.3 ± 1.3 b
		Without sugar	0.0 ± 0.0f	0.0 ± 0.0f	0.0 ± 0.0f	0.0 ± 0.0e	0.0 ± 0.0d	0.0 ± 0.0c	0.0 ± 0.0c	0.0 ± 0.0c
**Entrust 2SC 0.1×**	0.019531	With sugar	2.3 ± 1.5f	20.8 ± 9.2e	66.6 ± 8.7c	86.8 ± 3.5 b	100.0 ± 0.0a	100.0 ± 0.0a	100.0 ± 0.0a	100.0 ± 0.0a
		Without sugar	2.7 ± 1.8f	5.3 ± 2.3f	50.8 ± 8.8d	55.3 ± 8.2c	99.0 ± 1.0a	99.0 ± 1.0a	100.0 ± 0.0a	100.0 ± 0.0a
**Entrust 2SC 0.5×**	0.097656	With sugar	2.3 ± 1.5f	58.1 ± 4.4c	93.3 ± 3.9ab	99.0 ± 1.0a	99.0 ± 1.0a	100.0 ± 0.0a	100.0 ± 0.0a	100.0 ± 0.0a
		Without sugar	0.0 ± 0.0f	33.7 ± 6.3de	90.2 ± 2.9 b	98.0 ± 1.4a	98.9 ± 1.1a	100.0 ± 0.0a	100.0 ± 0.0a	100.0 ± 0.0a
**Entrust 2SC 1×**	0.195312	With sugar	20.7 ± 4.6d	81.3 ± 5.5ab	100.0 ± 0.0a	100.0 ± 0.0a	100.0 ± 0.0a	100.0 ± 0.0a	100.0 ± 0.0a	100.0 ± 0.0a
		Without sugar	6.7 ± 2.9ef	50.7 ± 6.4 cd	93.9 ± 1.7ab	98.8 ± 1.3a	100.0 ± 0.0a	100.0 ± 0.0a	100.0 ± 0.0a	100.0 ± 0.0a
**Actara 25WG 0.1×**	0.011442	With sugar	12.7 ± 5.1de	36.0 ± 6.4de	56.4 ± 9.0 cd	62.8 ± 8.5c	77.2 ± 5.8 b	97.8 ± 1.5a	98.8 ± 1.3a	100.0 ± 0.0a
		Without sugar	1.0 ± 1.0f	4.4 ± 2.1f	19.0 ± 4.3e	27.1 ± 4.4d	62.8 ± 5.7c	84.4 ± 3.7 b	89.7 ± 3.2 b	99.2 ± 0.8a
**Actara 25WG 0.5×**	0.057209	With sugar	62.6 ± 6.0 b	82.6 ± 10.6a	96.0 ± 2.1ab	96.7 ± 2.5a	100.0 ± 0.0a	100.0 ± 0.0a	100.0 ± 0.0a	100.0 ± 0.0a
		Without sugar	7.1 ± 3.7ef	34.7 ± 6.7de	63.7 ± 10.0 cd	87.7 ± 2.2 b	100.0 ± 0.0a	100.0 ± 0.0a	100.0 ± 0.0a	100.0 ± 0.0a
**Actara 25 WG 1×**	0.114418	With sugar	77.1 ± 6.0a	93.8 ± 2.2a	100.0 ± 0.0a	100.0 ± 0.0a	100.0 ± 0.0a	100.0 ± 0.0a	100.0 ± 0.0a	100.0 ± 0.0a
		Without sugar	40.1 ± 7.4c	69.4 ± 6.1bc	93.3 ± 3.2ab	96.0 ± 2.2a	98.5 ± 1.0a	98.5 ± 1.0a	100.0 ± 0.0a	100.0 ± 0.0a
***P-*value**			<0.001	<0.001	<0.001	<0.001	<0.001	<0.001	<0.001	<0.001
***F-*statistic (df = 13,126)**			36.8	35.2	50.7	102.2	216.3	481.0	561.1	2118.0

Means ± SE within each column followed by different letters are significantly different (*P *< 0.05). Fisher’s protected least significant difference (LSD) test was used in mean separation. The percentage mortality of earwigs was arcsine (square root (*x*)) transformed before analysis. Untransformed means are reported.

0.1×: 10% of label rate; 0.5×: 50% of label rate; and 1×: label rate.

a With sugar and without sugar tests consisted of hydrogel baits prepared in 25% (w/v) sucrose solution and water only, respectively.

### Field Experiment Using Hydrogel Bait With Added Sucrose

**Fig. 1.**
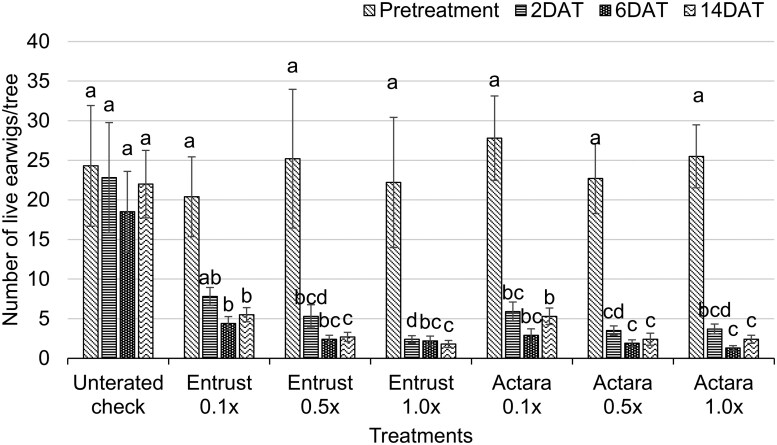
Mean ± SE number of live European earwigs per tree at three treatment concentrations: 0.1× (10% of the label rate), 0.5× (50% of the label rate), and 1× (label rate) across assessment dates (pretreatment, 2, 6, and 14 d after treatment). Bars sharing different letters within the same ­assessment day are significantly different (*P *< 0.05). Fisher’s protected least significant difference (LSD) test was used in mean separation. DAT, days after treatment (Dhungana and Gautam 2025a).

The number of live earwigs per tree prior to the treatment were similar across the treatments (*F *= 0.455; df = 6, 63; *P *= 0.839) (Fig. 1). After the treatment, we observed, the second day after treatment (2 DAT), the number of earwigs significantly reduced (*F *= 5.196; df = 6, 63; *P *< 0.001) in all treatments except for the 0.1× Entrust compared to the untreated check. On 6 and 14 DAT, all concentrations of both insecticide treatments showed a significant decrease (*F *= 5.196; df = 6, 63; *P *< 0.001) in earwig numbers compared to the untreated check. The numbers were lower in all concentrations of both insecticide treatments compared to the untreated check. Throughout the evaluation period, we observed that higher concentrations of both products tested resulted in fewer earwigs/tree (Fig. 1).

## Discussion

Our results show that insecticide-infused hydrogel baits, with or without added sucrose, can effectively reduce the European earwig population. Both Entrust (spinosad) and Actara (thiamethoxam) were effective for controlling European earwig, as found in previous reports in ant management (Milosavljević et al. 2024).

The laboratory bioassay provided critical insights into bait performance. The general lack of significant difference in mortality between choice and no-choice tests indicates that the hydrogel baits were highly attractive and/or palatable, even when an alternative food source was available. This is a critical attribute for a successful bait. Although the addition of sucrose to the hydrogel bait aimed to enhance its attractiveness when targeting sugar-feeding ants, our results showed no significant difference in mortality between treatments with and without sugar. This suggests that the hydrogel itself, likely providing essential moisture, may be a primary attractant (Day 2010). This finding has practical implications for simplifying bait formulation and minimizing cost. While research specifically comparing sugar-free hydrogels to sugary versions is limited, the report of Buczkowski (2020) suggests that removing sugar from hydrogels might further reduce the risk to pollinators, which are attracted to sugary substances.

The field trial confirmed a significant reduction in live earwigs just after two days of application for all treatments except the low rate of spinosad. A clear dose–response relationship was observed for both products, with higher concentrations yielding higher mortality, which aligns with standard toxicological principles (Matsumura 1985). This relationship provides both conventional and organic growers with operational flexibility, allowing them to select a rate that balances efficacy, cost, and considerations for non-target organisms. Moreover, hydrogel baits pose minimal risk to non-target insects and beneficial species (Buczkowski 2020).

The mode of action of these insecticides likely explains the observed mortality patterns. Spinosad, a fermentation-derived insecticide, acts primarily on the nicotinic acetylcholine receptors and gamma-aminobutyric acid receptors, causing rapid excitation of the insect nervous system (Salgado and Sparks 2005). Thiamethoxam, a neonicotinoid, is a potent agonist of the nicotinic acetylcholine receptor (Maienfisch et al. 2001).

In conclusion, insecticide-infused hydrogel baits represent a viable, targetable tool for suppressing European earwig populations in citrus when they pose an economic threat. This study provides a foundation for their use, showing effective mortality with minimal reliance on added sugars. Future research should focus on multi-season and large-scale field trials to confirm long-term efficacy and optimize bait station placement and density to maximize control while minimizing costs and non-target effects.
